# Potential Vaccines and Post-Exposure Treatments for Filovirus Infections

**DOI:** 10.3390/v4091619

**Published:** 2012-09-21

**Authors:** Brian M. Friedrich, John C. Trefry, Julia E. Biggins, Lisa E. Hensley, Anna N. Honko, Darci R. Smith, Gene G. Olinger

**Affiliations:** 1 United States Army Medical Research Institute of Infectious Diseases, Division of Virology, 1425 Porter Street, Frederick, MD 21702, USA; Email: brian.m.friedrich.ctr@us.army.mil (B.M.F.); john.c.trefry.ctr@us.army.mil (J.C.T.); julia.biggins@us.army.mil (J.E.B.); anna.honko@us.army.mil (A.N.H.); darci.smith1@us.army.mil (D.R.S.); 2 United States Food and Drug Administration (FDA), Medical Science Countermeasures Initiative (McMi), 10903 New Hampshire Avenue, Silver Spring, MD 20901, USA; Email: lisa.hensley@fda.hhs.gov (L.E.H.)

**Keywords:** filovirus, Ebola, ebolavirus, Marburg virus, marburgvirus, vaccines, post-exposure treatments, clinical trials, non-human primates, animal models

## Abstract

Viruses of the family *Filoviridae* represent significant health risks as emerging infectious diseases as well as potentially engineered biothreats. While many research efforts have been published offering possibilities toward the mitigation of filoviral infection, there remain no sanctioned therapeutic or vaccine strategies. Current progress in the development of filovirus therapeutics and vaccines is outlined herein with respect to their current level of testing, evaluation, and proximity toward human implementation, specifically with regard to human clinical trials, nonhuman primate studies, small animal studies, and *in vitro* development. Contemporary methods of supportive care and previous treatment approaches for human patients are also discussed.

## 1. Introduction

The family *Filoviridae* includes two accepted genera, *Ebolavirus *and *Marburgvirus*. The genus *Ebolavirus* includes five species (each represented by a single virus): *Zaire ebolavirus* (Ebola virus, EBOV), *Sudan ebolavirus* (Sudan virus, SUDV), *Reston ebolavirus* (Reston virus, RESTV), *Taï Forest ebolavirus* (Tai Forest virus, TAFV), and *Bundibugyo ebolavirus* (Bundibugyo virus, BDBV). The genus *Marburgvirus* includes a single species, *Marburg marburgvirus*, which has two members: Marburg virus (MARV) and Ravn virus (RAVV) [1,2]. In 1967, the first cases of filoviral infection were documented in three simultaneous outbreaks in West Germany and Yugoslavia. The virus responsible for the outbreaks was named “Marburg virus” after the German city of Marburg in which it was first recognized [3,4]. From documented instances of infection, it seems that the members of the filovirus genera may exist in quite opposite climates of Africa with marburgvirus infections occurring more frequently in the dry woodlands, while ebolavirus infections occur more frequently in rain forests [5]. More than 40 years of effort have focused on the search for the reservoir of these viruses in Africa, and while the search is still ongoing, recent evidence indicates that bats may be reservoirs for both marburgviruses and ebolaviruses [1,6,7,8,9,10,11]. However, the recent outbreak of RESTV in domestic pigs in the Philippines demonstrated the potential for animals other than primates and bats to be infected and potentially spread or amplify outbreaks [12]. 

Filoviruses are named for their long, filamentous shape which can been seen on the order of micrometers in length, while their width is more narrow (usually around 80 nm) with little fluctuation [13]. Contained within this filamentous virus is a single, 19-kb negative-sense RNA genome that encodes seven proteins [14,15]. The seven filoviral proteins are the glycoprotein (GP), the polymerase (L), the nucleoprotein (NP), a secondary matrix protein (VP24), the transcriptional activator (VP30), the polymerase cofactor (VP35), and the matrix protein (VP40) [16,17]. Homotrimers of the viral GP cover the surface of the virion, and this viral GP is believed to be the sole host attachment factor for filoviruses [6,18]. Candidates for filoviral receptor and co-factors include transferrin, DC-SIGN, TIM-1, and NPC1 [16,19,20,21,22]. After entry, filoviruses replicate their genomes and viral proteins in the cytoplasm using a RNA-dependent RNA-polymerase which is carried in with the virus. 

Wild-type filovirus infection has been associated with severe case fatality rates in humans, as high as 90% [15]. In humans, filovirus infection is characterized by an abrupt onset of flu-like illness, after an initial incubation period of 2–21 days. Following this initial illness, signs and symptoms of disease include anorexia, nausea, vomiting, chest pain, cough, edema, postural hypotension, neurologic complications, petechiae, and mucosal hemorrhage. There have also been several observed wild-type filovirus outbreaks among great apes in Africa that have demonstrated similarly high mortality rates [23]. In an effort to create cost and time-effective models of filoviral disease for the development of vaccines and therapeutics, small animals, such as mice and guinea pigs, are often used. However, these animals usually demonstrate significant resistance to wild-type filovirus infection, and only demonstrate mortality rates similar to primates when the filovirus in question has been adapted to the model species [24]. Due to the difficulties in evaluating wild-type filovirus infection in small animals and the generally high level of immune protection correlates derived from non-human primate (NHP) models of infection, therapeutics and vaccines are ultimately evaluated in NHP species for efficacy against filovirus. Of the NHP models available for filovirus study, rhesus and cynomolgus macaques have been the most highly characterized and utilized for therapeutic and vaccine development, respectively. However, the starting point of vaccine and therapeutic development remains small animal models due to the cost, ethical, and time-associated benefits [17]. This review will highlight the current research into filovirus vaccines and therapeutics. 

## 2. Current Treatments

### 2.1. Supportive Care

The current clinical standard for filoviral infection is supportive care as there are currently no FDA-approved treatment strategies. Supportive care consists of oral fluid rehydration, oral medication, nutritional supplementation, and psychosocial support [25]. Nasogastric feeding tubes and i.v. administration of both fluids and medication are increasingly considered supportive care where possible during outbreak scenarios to prevent dehydration and facilitate support of blood pressure [25,26]. However, given the limited equipment and laboratory support during outbreaks, care must be taken to prevent overaggressive fluid administration [27]. Fluid replacement was evaluated briefly in rhesus macaques, and while there was no significant benefit to survival, a less severe renal compromise was observed [28]. While supportive care may (or may not) reduce the overall case fatality rate in humans, the true impact of simple interventions such as fluid management has yet to be fully evaluated and the potential for benefit in combination with direct antiviral measures has yet to be assessed [29]. 

### 2.2. Immunotherapy

Treatment of filovirus infection with passive transfer of antibodies is an attractive therapy. While there have been conflicting results *in vitro*, in animals, and in humans, recent breakthroughs have solidified the potential for this strategy of intervention. In addition, there have been a number of immunotherapies developed for other agents, such as respiratory syncytial virus (RSV), which can provide potential roadmaps or precedence to facilitate the advancement of these products through the necessary regulatory hurdles [30,31]. Transfer of immune serum for the treatment of filovirus infection in humans has previously been attempted. However, interpretation of these results has been cautious due to the study conditions as well as the uncertainty of the disease stage at which the individuals were treated [6]. As a result, much attention has focused on animal studies evaluating candidate products. While many of the early studies in mice and guinea pigs were successful, these successes did not translate to NHP studies and tempered the enthusiasm for further evaluation of candidate products. [6,32,33,34,35]. When similar passive transfer strategies were attempted in NHPs, viremia onset and outward signs of disease were reduced, but the treatment did not affect survivorship [36,37,38]. In addition, the suggestion that antibodies could enhance filovirus infections *in vitro* caused further concern [39,40,41]. 

However, recently a series of experiments have made researchers, developers, and funding agencies reconsider the potential of this category of products for filoviruses. Both polyclonal and monoclonal passive therapies have been shown to be efficacious in rodents for filovirus infection [42,43,44]. Furthermore, evidence of enhancing antibodies exists in the antibody response to EBOV [38]. More recent studies have demonstrated protection in macaques with polyclonal and monoclonal passive therapy [45,46,47]. These sources of monoclonal antibodies have ranged from murine monoclonal antibodies to recombinant-derived cloned human monoclonal antibodies from survivors of filovirus infection [37,43]. 

Development of new antibodies to be used for post-exposure treatment is on-going. In one study, an antibody (13F6) targeting the EBOV GP mucin-like domain was generated and subsequently shown to protect 100% of mice against a lethal EBOV challenge when given 2 days post-exposure [44]. This antibody was then modified to generate h-13F6, a human recombinant antibody. This human recombinant antibody also significantly protected mice against a lethal challenge of EBOV [48]. In another method, a recombinant VSVΔG/EBOVGP was used to generate a total of 8 monoclonal antibodies which were subsequently characterized. All 8 monoclonal antibodies improved survival rate of mice (33%–100%) against a high-dose lethal challenge by mouse-adapted EBOV [49]. Another antibody, KZ52, was isolated from the bone marrow of a human survivor of EBOV infection and is specific for the complex of GP1and GP2 [50]. KZ52 neutralized EBOV *in vitro* and offered protection from lethal EBOV challenge in a rodent model [43], but was non-protective in NHPs [37]. 

## 3. Vaccines

### 3.1. Vaccines in Human Clinical Trials – Summarized in Table 1

#### 3.1.1. DNA Vaccines

The first clinical trials involving filovirus vaccines were based off of plasmids expressing EBOV NP and GP as well as SUDV GP [51]. This strategy proved safe in 27 subjects involved with phase I testing. However, the prime/boost DNA vaccine strategy covering four separate plasmid doses administered three times each was ineffective at creating durable immunity as evidenced by the near non-existent antibody titer in these subjects after 1 year [51]. While clinical trials with this vaccine have halted, it may be possible that this strategy can supplement another vaccine technology in a prime/boost capacity. 

**Table 1:** Vaccines in Clinical Trials or Effective in Non-human Primates. Comparison of current vaccine candidates at the highest levels of development, either in human clinical trials or those that have shown promise in non-human primates (NHPs). Also listed are the afforded levels of immunization/protection, the type of vaccine used to induce immunity and the vaccination paradigm used to achieve the listed results.

Vaccine	Type	Mechanism	Species Tested	Efficacy	Strategy
**DNA Vaccine**	DNA vaccine	Adaptive Immune Response	Cynomolgus macaque	100% EBOV	3 i.m. injections, 4 weeks apart
**Ebola rAd5 vaccine**	Vector-based vaccine	Adaptive Immune Response	Cynomolgus macaque	100% EBOV	Single i.m. injection
**CAdVax-based EBO7 vaccine**	Vector-based vaccine, blend of 4 vectors expressing 5 different genes	Adaptive Immune Response	Cynomolgus macaque	100% EBOV	2 i.m. injections, 9 weeks apart
				100% SUDV	
				100% MARV	
**VSVΔG/EBOV-GP vaccine**	Vector-based vaccine, can be single vector or multiple vector blend, replication competent	Adaptive Immune Response	Rhesus macaque	100% EBOV	Single i.m. injection
				100% SUDV	
				100% TAFV	
				75% BDBV	
				100% MARV	
**VEE Replicon Particle (VRP) vaccine**	Vector-based vaccine, single round replication	Adaptive Immune Response	Cynomolgus macaque	100% MARV	3 i.m. injections, 4 weeks apart
**HPIV-3 vaccine**	Vector-based vaccine, replication competent	Adaptive Immune Response	Rhesus macaque	100% EBOV	2 i.n./i.t. inoculations, 4 weeks apart
**NDV-GP**	Vector-based vaccine, replication competent	Adaptive Immune Response	Rhesus macaque-immune response evaluation (not challenged)	Less immunogenic than HPIV-3 but could augment HPIV-3 in prime/boost strategy	2 i.n./i.t. inoculations, 4 weeks apart
**VLP**	Non-replicating virus particle vaccine	Adaptive Immune Response	Cynomolgus macaque	100% EBOV	3 i.m. injections, 6 weeks apart
				100% MARV	

#### 3.1.2. Ebola rAd5 Replication Defective Vaccine

While no vaccines or therapeutics are currently licensed for use by the FDA, phase I clinical trial safety tests have been performed on one particular platform for an EBOV vaccine. This vaccine is based on a replication deficient, recombinant adenovirus serotype 5 (rAd5) vector genetically engineered to carry the genes for EBOV glycoprotein (GP (Z)) and SUDV glycoprotein (GP (S/G)). As a common human pathogen, this vector vaccine utilized the broad-tropism of the adenovirus vector to infect cells and once inside the inserted ebolavirus glycoproteins are expressed. Upon expression of these inserted ebolavirus genes, the host immune system will recognize them as foreign and mount a response against them. The advancement of this vaccine technology to phase I trials manifested from its ability to provide 100% protection among cynomolgus macaques vaccinated 28–35 days prior to challenge and its ability to generate potent humoral and cell-mediated immune responses [52,53]. 

In the clinical trial participants remained asymptomatic after a single vaccination with either 2 × 10^9^ or 2 × 10^10^ viral particles [54]. Furthermore, for both doses of the vaccine significant antibody titers were observed at 4 weeks post-vaccination with 100% and 55% of participants receiving 2 × 10^10^ viral particles being positive for GP (S/G) and GP (Z), respectively. Significant antibody titers were observed again at 48 weeks post-vaccination and, while decreased from 4 weeks, demonstrated the potential durability of this vaccine over time [54]. T-cell activation was also examined for these individuals and found to directly correlate with the dose administered, but to a lesser extent than the previously mentioned antibody response. CD4^+^ activation was observed with greater frequency than CD8^+^ activation in those receiving the vaccine. Importantly, these results were obtained in the context of significant pre-existing immunity to Ad5 as pre-entry evaluation of antibody titers revealed that 50% of participants were positive against Ad5, showing that pre-existing immunity to Ad5 still resulted in protection against EBOV [54]. While this study shows great promise, further development and additional studies in NHPs and humans are needed. 

### 3.2. Vaccines Effective in NHPs – Summarized in Table 1

#### 3.2.1. Other Adenovirus Vector Vaccines

In addition to the adenovirus platform in clinical trials, additional variations of the rAd5 vaccine are also in development and have been evaluated in NHP models. Based on the adenovirus vector platform, the complex adenovirus (CAdVax) technology substantially increased the genetic payload capacity of the vector, up to 7 kB. Additionally, this strategy involved the blending of four separate vectors expressing the glycoproteins of EBOV, SUDV, and MARV along with the nucleoproteins of EBOV and MARV. When administered in a prime/boost strategy, this technology offered 100% protection against EBOV, SUDV, and MARV [55]. Another variation of the CAdVax system designed to express modified EBOV glycoprotein and SUDV glycoprotein was effective in protecting against both parenteral and aerosol challenge when administered in a prime/boost strategy [56]. Both implementations of the CAdVax technology demonstrated significant antibody titers. 

Further improvement upon the adenovirus-based EBOV vaccine technology is ongoing. Richardson *et al. *reformatted the genetic insert for the vector which included the addition of a cytomegalovirus (CMV)-chicken β-actin hybrid promoter, optimized codons and a consensus Kozak sequence [57]. These improvements led to three- to seven-fold increases in EBOV glycoprotein expression. Neutralizing antibody titers were found at doses as low as 10^4^ infectious forming units (IFU) with comparable titers requiring 10^7^ IFU of the unmodified vaccine in mice. These modifications demonstrated 100% protection of mice at doses two orders of magnitude lower than the unmodified vaccine. Interestingly, at 30 min post-challenge, the modified Ad-CMVZGP/Ad-CAGoptZGP offered 100% protection compared to the 22% protection of mice offered from the original vaccine [57]. This vector format has also recently showed promise when administered sublingually in mice, therefore eliminating the complexities of parenteral administration such as the necessity for sterile tools, aseptic chemicals, and the risks of potential blood-borne pathogen exposure [58]. 

While this adenovirus vector vaccine technology is promising, demonstrations that pre-existing immunity to the Ad5 vector depressed the desired immune response may impede its implementation. In efforts to circumvent issues of pre-existing immunity to Ad5, Geisbert *et al.* sought out a less prevalent serovariation [59]. In their study, a heterologous prime/boost strategy with recombinant adenovirus serotypes 26 and 35 carrying GP (Z) and GP (S/G) demonstrated complete protection among NHPs. Each of these vectors was capable of stimulating humoral and cell-mediated immune responses in the context of NHPs pre-vaccinated with rAd5 as evidenced by antibody titers reaching an order of magnitude above those achieved in rAd5 vaccinated subjects (1:32,000 compared to 1:6,800), and CD8^+^ intracellular cytokine staining was 4.7-fold greater among heterologous prime/boosted subjects (0.41% compared to 0.09%) [59].

#### 3.2.2. Rhabdovirus Vector Vaccines

Rhabdoviruses have recently offered unique vaccine platforms to generate both genus/species specific immunity as well as potential for cross-protective immunity for filoviruses. For example, based on an attenuated recombinant vesicular stomatitis virus (rVSV), the replication-competent virus expresses the glycoprotein of the target filovirus in place of its wild-type membrane glycoprotein. As this virus is primarily an agricultural pathogen, pre-existing immunity interfering with the desired immune response and subsequent protection is unlikely [60]. Several studies have begun to address the safety of the filovirus VSV platforms. Evaluation of this platform in immunocompromised NHPs has suggested that this technology may be safe among similarly immunocompromised humans [61]. Further encouragement for the safety of this live-attenuated vaccine came recently from Mire *et al.* who showed that EBOV and MARV rVSV showed no signs of neurovirulence associated with VSV [62].

The utility of the VSV-based vaccine for protection against filoviral hemorrhagic fever was highlighted by Geisbert *et al.* [63]. Using a blended vaccine consisting of equal amounts of three different VSV vectors each carrying the EBOV, SUDV or MARV glycoprotein, they were able to generate 100% protection of NHPs against challenges with EBOV, SUDV, TAFV, and MARV with no observed ill effects from this replication-competent vaccine. Of all vaccinated NHPs, only one showed signs of viremia as assayed by RT-PCR. Each of the vaccinated NHPs also demonstrated elevated antibody responses after vaccination, with titers ranging from 1:32 to 1:100 for all three glycoprotein components of the blended vaccine [63]. 

In addition to providing such high levels of protection as a prophylactic vaccine strategy, the VSV-based technology has demonstrated post-exposure protection for both EBOV and MARV when administered via intramuscular (i.m.) injection [64]. When MARV-rVSV was administered i.m. 20–30 min post-challenge with MARV, 100% of NHPs survived. In this study viral RNA was observed in the blood on day three post-challenge when assayed by RT-PCR, but active virus was unobservable by traditional plaque assay. Clinical chemistry results demonstrated that these surviving NHPs experienced significant rises in aspartate aminotransferase, gamma-glutamyl transferase, total bilirubin, and blood urea nitrogen indicating that, while protective, the post-exposure treatment did not completely prevent typical pathogenic events associated with MARV infection. Similar experiments demonstrated SUDV-rVSV delivered 20–30 min after challenge offered 100% protection [65]. Post-exposure protection for EBOV-rVSV was less effective at 20–30 min but still afforded 50% protection to eight NHPs [66]. As a post-exposure treatment, EBOV glycoprotein rVSV was used recently 48 h after a suspected human exposure via needlestick in the laboratory. While there is no direct evidence the laboratory worker was indeed exposed, that person survived the experience with no discernible sequelae from the treatment outside of a transient fever occurring 12 h after an injection of 5 × 10^7^ plaque forming units (PFU) [67]. 

Also of note for rhabdovirus-based filoviral vaccines was a recent report that generated dual immunity for both EBOV and rabies virus infection. EBOV GP was efficiently expressed from an attenuated vaccine used for wildlife against rabies virus in place of the wild-type rabies envelope glycoprotein, G [68]. This vaccine vector was capable of inducing protective immunity to EBOV infection as well as to rabies virus infection in both live-attenuated format and β-propriolactone inactivated vaccine. Neurovirulence of the recombinant vector was unobserved in suckling mice when compared to the unaltered vaccine [68]. This versatility offers increased storage options with an inactivated vaccine as well as the opportunity to vaccinate for each disease where they are both endemic.

#### 3.2.3. Venezuelan Equine Encephalitis Virus-Based Replicon Particles (VRP)

Alphavirus particle-based vaccines also provide high levels of protection to NHPs against lethal filovirus challenge. These vaccines for EBOV, SUDV, and MARV are composed of Venezuelan equine encephalitis virus (VEEV)-based replicon particles (VRPs) that express the viral glycoprotein of interest [69]. Replicon particles are replication-defective, single-cycle vectors which cannot spread from cell-to-cell. The VRPs are composed of an attenuated VEEV replicon that contains VEEV non-structural genes and the filovirus glycoprotein. VRPs are generated when the replicon is co-transfected into cells with helper plasmids containing the VEEV structural genes. The resulting single-cycle propagation defective particles are then administered to the appropriate animal model for efficacy testing [69,70]. This technology offers several advantages, including high expression levels of heterologous genes, dendritic cell tropism, induction of robust cellular and antibody immune responses, rapid construction into single and multivalent vaccines, and relative resistance to anti-vector immunity [69,70]. 

*In vivo* studies with the MARV VRP offered the first demonstration of a fully protective filovirus vaccine. NHPs exhibited 100% survival after vaccination with 10^7^ focus forming units (FFU) of VRP in three consecutive doses spaced at 28 day intervals prior to challenge with MARV [71]. This protection was offered when the VRPs were constructed to express GP alone or GP + NP; however, the NHPs vaccinated with NP alone all exhibited clinical symptoms of illness and only two out of three survived the challenge. Substantial antibody titers were found in each of the vaccinated NHPs. Additionally, no conspicuous elevations in clinical chemistries were observed in NHPs throughout the experiment. Experiments performed on mice and guinea pigs supported the ability of VRPs expressing GP to mediate complete protection from lethal MARV and EBOV challenge [71]. In mice, adoptive transfer of CD8^+^ cells, but not CD4^+^ cells or passive antibody transfer, from VRP-NP-immunized mice was protective, suggesting this vaccine may be most protective by stimulating the host cell-mediated immune response [72]. Additionally, adoptive transfer of CD8^+^ T-cells after activation via specific EBOV peptides provided mice complete protection indicating a mechanism for VRP-based immunity [73]. Recent studies indicate that a VRP-based vaccine is fully protective in cynomolgus macaques against EBOV, SUDV, and MARV parenteral and aerosol virus challenges (unpublished observations, Olinger).

#### 3.2.4. Paramyxovirus-Based Vaccines

Paramyxovirus-based vectors for vaccination against filoviral threats have recently demonstrated the capacity to protect NHPs from infection and stimulate strong immune responses. Paramyxoviruses have a natural tropism for the respiratory tract and, as filoviruses are both emerging diseases and potential weaponized threats, the idea of targeting vaccines to this area is ideal. Two candidates for this category of vaccines have been investigated to date: human parainfluenza virus 3 (HPIV-3) and Newcastle disease virus (NDV). Of these two systems, the HPIV-3 system has been evaluated in NHP models of EBOV infection. Combinations of EBOV GP alone, EBOV GP + NP, and EBOV GP + human granulocyte macrophage colony stimulating factor (GM-CSF) were inserted into the genome of HPIV-3. Each of these vaccine vectors was used to vaccinate NHPs both intranasally (i.n.) and intratracheally (i.t.) as initial studies offered complete protection of guinea pigs vaccinated via the respiratory route [74]. At least one NHP in all three vaccine groups receiving 4 × 10^6^ TCID_50_ (median tissue culture infective dose) of their respective vector displayed clinical signs of illness after challenge during the study. Each group held two NHPs and out of the three groups only one vaccinated animal from the EBOV GP + NP succumbed to the disease. Immune responses from these subjects, prior to challenge, revealed antibody titers ranging from 1:400 to 1:1,600 [75]. By manipulating dose and administration strategies, Bukreyev *et al.* were able to achieve complete protection of NHPs after two successive doses of 2 × 10^7^ TCID_50_ given at day zero and again at day 28 with challenge occurring on day 67 [75]. The two-dose strategy produced IgG titers ranging between 1:1,600 and 1:25,600, much higher than the single dose. Each of these experiments highlights the potential of the HPIV-3 platform for EBOV vaccination but the known prevalence of pre-existing immunity to HPIV-3 in humans could hinder the generation of targeted immunity [76]. To address these concerns, Bukreyev *et al.* compared the immunogenicity of EBOV GP expressing HPIV-3 vector among naïve and pre-immune NHPs [77]. In these experiments EBOV-specific IgG levels were substantially decreased among HPIV-3 pre-immune NHPs; however, this hindrance was overcome when the NHPs were vaccinated with two doses of recombinant vector which was previously shown to offer complete protection against EBOV challenge [77]. 

In efforts to diversify the paramyxovirus-based vectors and avoid issues surrounding the pre-existing immunity found for HPIV-3, a new vector design based on NDV was established. NDV is an avian paramyxovirus that infects the respiratory tract. This virus has been shown to be highly attenuated in NHPs due to natural host restriction processes [78]. Additionally, this vector system has proven successful as a vaccine platform for severe acute respiratory syndrome-associated coronavirus (SARS-CoV) and influenza H5N1 in NHPs [79]. Although this system has yet to be evaluated in the context of NHP models of EBOV infection and disease, it was recently shown to be immunogenic in NHPs. Single vaccination with NDV expressing EBOV GP produced lower titers than the HPIV-3 platform, demonstrating this vector is less immunogenic; however, in a homologous prime/boost vaccination strategy EBOV-specific mucosal IgA levels reached those similar to the HPIV-3 homologous prime boost vaccination strategy [80]. IgG specific for EBOV did not reach levels comparable to the previous HPIV-3 platform. These reports support the potential use of paramyxoviruses as vaccine candidates, but further examination of immunostimulatory effects and pre-existing immunity will require investigation. 

#### 3.2.5. Virus-Like Particles (VLPs)

The VLP technology works by generating non-replicating virus particles that do not contain any filoviral genetic material. Immune responses generated in response to exposure to VLPs are derived from the filoviral protein shell that is the VLP itself. VLPs are constructed through the matrix protein VP40’s ability to drive the budding process. By simple transfection and expression of VP40 into target cells, filamentous structures can be generated [81]. Co-expression of additional filoviral proteins, such as GP and NP, can dramatically enhance and stabilize the production of VLPs from target cells [82]. When traditional target/production cells were swapped out for insect cells and filoviral protein expression was driven from a baculovirus vector, VLPs were generated and found to have filamentous structures resembling wild-type virus. VLPs have demonstrated immunogenicity and the ability to protect NHPs from lethal challenge. The first such study involving NHPs utilized the baculovirus-produced VLPs containing EBOV GP, NP, and VP40 [83]. Five cynomolgus macaques were vaccinated three times at 42-day intervals with 250 µg of VLP with RIBI adjuvant. After the full vaccination schedule, 100% survived a lethal EBOV challenge, and there was a three- to ten-fold increase in EBOV specific antibodies which possessed an 80% plaque reduction at titers between 1:20 and 1:160 [83]. Additionally, these antibodies were shown to have both compliment-mediated and antibody-dependent cell-mediated cytotoxic properties [83]. Using this same VLP technology, Swenson *et al.* were able to show a similar effect for MARV. Three i.m. injections of 1 mg of VLP in 0.1 ml of QS-21 adjuvant spaced 42 days apart offered 100% protection against MARV and RAVV; however, one animal challenged with RAVV did exhibit slight morbidity [84]. All animals had no detectable viremia as determined by plaque assay at days 0, 3, 5, 7, 10, 14, and 21 post-exposure. Additionally, two injections of the VLPs correlated with a peak in homologous antibody titer while three injections correlated to peak heterologous antibody titer [84]. The ability of VLPs to generate protective immunity against filoviral challenge has been demonstrated in guinea pigs as well [85]. 

### 3.3. Vaccines Effective in Small Animal Models – Summarized in Table 2

#### 3.3.1. Virus-Like Particles (VLPs) Derived from Baculovirus Vectors

Immunogenic virus-like particles (VLPs) of EBOV and MARV can be easily generated in mammalian systems. EBOV-like particles (VLPs) can be efficiently generated in mammalian cells after expression of VP40 alone, but other filovirus proteins can be co-expressed as well [85,86,87]. Baculovirus-derived VLPs have also been successfully generated in insect cells and stimulated both cellular and antibody immune responses against hepatitis E virus, human papilloma virus, rotavirus, and simian immunodeficiency virus [88,89,90,91]. Several groups have made and tested baculovirus-generated filovirus-VLPs as vaccines in small animal models. Warfield *et al.* generated Ebola-VLPs (eVLP) and Marburg-VLP (mVLP) containing VP40, NP, and GP. This vaccine had up to 100% survival following a lethal infection in mice (vaccine dose-dependent) [85]. Sun *et al.* produced an eVLP containing VP40 and GP. This vaccine was also up to 100% effective following a lethal EBOV infection in mice (vaccine dose-dependent) [92,93]. 

#### 3.3.2. EBOVGP-Fc Fusion Protein

Many reports document the ability of filoviral GP to act as a potent immunogen, and as such, viral vectors are a popular method of vaccinating through the expression of GP by the host and subsequent immune recognition. However, a recent report sought to utilize GP itself as the vaccination agent. In order to efficiently produce the protein, an expression vector was constructed such that the Fc portion of a human IgG was fused to EBOV-GP [94,95,96]. This GP-Fc fusion protein induced both cell-mediated and humoral immune responses, and mice vaccinated with ZEBOVGP-Fc demonstrated 90% protection against a lethal EBOV challenge. [97]. While further studies are required, these results show that vaccination with EBOVGP-Fc alone is effective against a lethal EBOV infection, and thus fusion proteins could potentially be used as an effective vaccine against filoviruses [97].

**Table 2:** Vaccines Effective in Small Animal Models. Comparison of current vaccine candidates at the small animal model level of development. Also listed are the afforded levels of immunization/protection, the type of vaccine used to induce immunity and the vaccination paradigm used to achieve the listed results.

Vaccine	Type	Mechanism	Species Tested	Efficacy	Strategy
**Ebola_VP30/Baculovirus**	Virus-like particle (VLP)	Adaptive Immune Response	Mice	Drug dependent-up to 100%	Multiple i.m. injections multiple boosts
**EBOV-GP-Fc fusion protein**	Fusion protein/ subunit vaccine	Adaptive Immune Response	Mice	Up to 90%	4 i.p. injections, ~3 weeks apart
**Nicotiana Benthamiana**	Subunit vaccine	Adaptive Immune Response	Mice	High specific antibody titer	4 s.c. injections, 3 weeks apart
**mCMV/EBOV-NPCTL **	CMV-based vaccine	Adaptive Immune Response	Mice	Drug dependent-up to 100%	2 i.p. injections, 4 weeks apart
**HPIV3/ΔF-HN/EboGP**	Vector-based vaccine, replication competent	Adaptive Immune Response	Guinea Pigs	100% EBOV	1 i.n. inoculation, 25 days prior to infection

#### 3.3.3. Nicotiana Benthamiana-Produced Immune Complex Subunit Vaccine

The use of plants to produce vaccine antigens and antibodies has been demonstrated previously and is attractive for several reasons, including low manufacturing cost, efficient production, minimal risk of contamination with human pathogens or toxins, and the fact that plants have similar secretory pathways and endosomal systems as mammalian cells [98,99,100,101]. Recently, a bean yellow dwarf virus (BeYDV)-derived replicon system was developed and shown to efficiently produce antibodies against EBOV in *Nicotiana benthamiana* leaves [100]. Poolcharoen *et al.* used this system to develop and fuse EBOV-GP1 to the C-terminus of an IgG heavy chain [102]. These EBOV immune complexes (EIC) were then used as a vaccine to immunize mice. Serum antibody response tests showed that this EIC was highly immunogenic in mice and produced antibody levels similar to mice protected from a lethal EBOV challenge [102]. While antibody titers alone do not fully correlated with protection from lethal challenge, there is potential for further development of this vaccine. 

#### 3.3.4. MCMV/EBOV-NPCTL

A replicating vaccine that could spread through the target population after initial inoculation would be an attractive, alternative approach to filovirus development. This approach could provide high coverage with minimal initial vaccinations. A CMV-based vaccine would allow for this type of spread [103]. CMV, a member of the family Betaherpesvirinae, induces a high “effector” memory T-cell (T_EM_) response before establishing a low-level persistent infection [104,105,106]. This high immunogenicity makes CMV a good potential vaccine vector, and the CMV vector has been previously shown to be effective as a vaccine for SIV in rhesus macaques [104,107]. Tsuda *et al.* constructed a mouse CMV vector expressing a CD8^+^ T cell epitope from the nucleoprotein (NP) of EBOV (MCMV/EBOV-NPCTL). This vaccine induced high levels of EBOV-specific CD8^+^ T cells, and subsequently, protected 100% of mice against a lethal EBOV challenge. This shows a proof-of-concept for CMV as a potential vaccine vector for EBOV [103].

#### 3.3.5. HPIV3/ΔF-HN/EboGP

As discussed previously, paramyxovirus-based vectors have demonstrated the capacity to induce strong immune responses and protect animals from infection. As such, a chimeric HPIV3 was developed where all the HPIV3 surface markers/receptors were deleted and replaced with EBOV-GP [108]. This chimeric virus can be amplified and recovered easily. Additionally, this chimeric virus has 2-fold greater incorporation of EBOV-GP into the virion due to the lack of competition with HPIV3 surface proteins [108]. Testing in guinea pigs showed that HPIV3/ΔF-HN/EboGP is highly attenuated, as compared to both HPIV3 and HPIV3/EboGP, and immunogenic, as 67% developed neutralizing antibodies against EBOV. Finally, a 4 × 10^6^ PFU i.n. inoculation of HPIV3/ΔF-HN/EboGP was able to protect 100% of guinea pigs against a lethal EBOV challenge [108]. 

## 4. Post-Exposure Treatments

### 4.1. Post-Exposure Treatments in Human Clinical Trials – Summarized in Table 3

#### 4.1.1. rNAPc2

Severe coagulation disorders are one of the most prominent features of filoviral infection. In the event of a breach in vascular integrity a strict balance of pro- and anti-coagulant host factors must be maintained to successfully clot the breach and to prevent too much or too little clot formation. In the instance of filovirus infection, sustained microvascular injury in effected organs results in host coagulation inhibitor depletion which results in disseminated intravascular coagulation (DIC). In DIC, tissue factor (TF), a clotting factor normally present on cells not exposed to blood, complexes with circulating factor VII leading to clot formation and fibrin deposition through the extrinsic pathway [109,110]. As numerous studies have demonstrated a clear link with DIC and resultant organ failure, Geisbert *et al*, sought to eliminate potential TF pathogenesis during filovirus infection by using recombinant nematode anticoagulant protein c2 (rNAPc2) [111]. They demonstrated that rNAPc2, an inhibitor of the TF pathway, provided partial post-exposure protection to rhesus macaques during filovirus infection [111]. Previous studies with rNAPc2 have already gone through phase II trials in orthopedic surgery [112] and coronary revascularization [113]. Geisbert *et al.* showed a 33% survival rate, in addition to a 3.4-day increase in mean time-to-death, for EBOV-infected rhesus macaques and a 17% survival rate, with a 1.7-day increase in mean time-to–death, in MARV-infected rhesus macaques, when treated with rNAPc2 post-exposure [111,114]. In a normally 100% lethal model of filovirus infection, rNAPc2 demonstrated a clear benefit to survival as an increase in the mean time-to-death was observed. rNAPc2 has completed phase II clinical trials with a good safety record. 

**Table 3:** Post-Exposure Treatments in Human Clinical Trials or Effective in Non-human Primates. Comparison of current drug candidates at the highest level of development, either in human clinical trials or those that have shown promise in NHPs. Also listed are the afforded levels of protection in NHPs, the type of drug used to induce immunity and the dosing paradigm used to achieve the listed results.

Treatment	Type	Mechanism	Species Tested	Efficacy	Strategy
**rNAPc2**	Recombinant protein	Blocks TF:FVIIa mediated activation of factor X	Rhesus macaque	33% (EBOV)17% (MARV)	Daily s.c. injection of 30 µg/kg
**RNA Interference**	PMOs	Targets viral mRNA to block transcription	Rhesus macaque	Drug dependent-may be up to 100% immediately post-exposure	Daily s.c./i.p. or i.v. injections of 40 mg/kg
**rhAPC**	Recombinant protein	Anti-thrombotic: cleaves and inhibits coagulation cofactors FVIIIa and FVa	Rhesus macaque	20% (EBOV)	Daily s.c. injection of 30 µg/kg

#### 4.1.2. Phosphorodiamidate Morpholino Oligomers

Phosphorodiamidate morpholino oligomers (PMOs) exert their anti-translation effects through steric hindrance of the translation machinery. This steric hindrance is possible due to a morpholino group, similar to a ribose base in RNA, and a methylene phosphorodiamidate linking moiety that physically bind to mRNA and prevent the translation machinery from accessing the mRNA [115]. Once the antisense PMOs bind to their target mRNA, they are highly stable and highly soluble which would allow high levels of translation inhibition and predictably low levels of potential cytotoxicity [115,116,117,118]. PMOs have previously demonstrated effective antiviral activity against coronaviruses and flaviviruses [119,120]. Swenson *et al.* initially utilized PMOs targeting EBOV VP24 and EBOV VP35 to highly protect mice and guinea pigs against a lethal challenge with EBOV and MARV [121,122]. Subsequently, AVI-6002 (a combination of PMOs against both EBOV VP24 and VP35) and AVI-6003 (a combination of PMOs against both MARV VP24 and NP) were developed and tested in a NHP post-exposure scenario. These PMOs, delivered 30–60 min post-exposure, protected >60% of rhesus macaques against lethal EBOV infection and 100% of cynomolgus macaques against MARV infection [123]. Both the ease of controlled manufacture and their efficacy in NHP models to combat filovirus infection, PMOs represent a viable therapeutic strategy [123]. Currently, AVI-6002 and AVI-6003 are in phase I clinical trials. 

### 4.2. Post-Exposure Treatments Effective in NHPs – Summarized in Table 3

#### 4.2.1. Recombinant Human Activated Protein C

Ebolavirus disease (EVD) and severe sepsis (or septic shock) share many clinical features including fever, hypotension, increased production of tissue factor, elevated levels of nitric oxide, and elevated levels of D-dimers [124,125,126]. In addition, the most prominent and consistent finding in severe sepsis is severe protein C deficiency [127,128]. It was shown that treatment of patients with severe sepsis with recombinant human activated protein C (rhAPC) resulted in improved survival [129]. Later experiments in NHPs demonstrated that EBOV infection results in rapid reduction of circulating protein C levels [111,130]. Therefore, it was tested if treatment with rhAPC could protect against lethal EBOV infection in rhesus macaques. Fourteen rhesus macaques were infected with a lethal dose of EBOV; eleven were then treated with IV rhAPC 30–60 min after challenge, continuing for 7 days. All control animals died on day 8 post-exposure; however, 2 of the 11 rhAPC-treated animals survived (~20% survival). Additionally, the mean time-to-death for rhAPC-treated animals was 12.6 days, which is significant compared to the 8.3 days observed in placebo and historical controls [125]. This product was pulled as a single post-exposure treatment, but given that this intervention is not directly targeting the virus, there may be additional merit in assessing this product in conjunction with a direct antiviral.

#### 4.2.2. RNA Interference and Stable Nucleic Acid Lipid Particles

RNA interference (RNAi) represents a powerful, naturally occurring biological strategy for inhibiting gene expression. RNAi interferes with the translation of mRNA to protein products by either sterically blocking mRNA or by triggering RNase H-mediated cleavage of the DNA/RNA duplex, resulting in inhibition of gene expression [122]. For many years RNAi as demonstrated clear efficacy in preventing viral replication *in vitro* against a number of viruses, including coxsackieviruses, hepatitis B virus (HBV), hepatitis C virus (HCV), herpesviruses, human immunodeficiency virus 1 (HIV), human papillomavirus, RSV, influenza A virus, lymphocytic choriomeningitis virus, polioviruses, and SARS-CoV [131,132,133,134]. Fowler *et al.* demonstrated that small-interfering RNA (siRNA) downregulation of various MARV mRNA transcripts was able to significantly decrease viral protein production and subsequent viral release in cell culture [135]. Unfortunately, efficient delivery vehicles providing effective drug targeting and stability have hindered the application of RNAi in the clinical setting. However, recent developments in the field of nanotechnology have made nanoparticles the solution to increasing pharmacokinetic profiles for RNAi therapies [136].

Additionally, Tekmira, Inc. developed proprietary lipid encapsulation as a means of improving the pharmacology of siRNA targeting the Ebola RNA polymerase L protein, as demonstrated by Geisbert *et al.* [137,138]. To efficiently deliver the siRNA to target cells, a mixture of lipids forming a bilayered liposome, or stable nucleic acid-lipid particles (SNALP), was designed. The SNALP ensures cell entry by preferential fusing with the endosomal membrane upon exposure to the decreasing pH of the endosome. The SNALPs were further modified by conjugation with polyethylene glycol (PEG) ensuring stability and efficient delivery of the siRNA payload by neutralizing surface charges and presenting a hydrophilic exterior. This encapsulation was initially demonstrated to significantly increase the stability, half-life, and effectiveness of siRNA directed against HBV [139]. The SNALP-encapsulation of siRNA targeting the EBOV L protein was initially shown to completely protect guinea pigs when administered shortly after a lethal EBOV challenge [137]. This treatment was then assessed for efficacy in rhesus macaques. SNALP-encapsulated siRNAs targeting EBOV L polymerase, VP24, and VP35 were given to rhesus monkeys either four or seven times following a lethal challenge of EBOV. Two of the three monkeys given four doses survived lethal infection, while all four monkeys given seven doses survived infection [138]. The enhanced survivorship among the SNALP-treated group highlights the efficacy of this potential therapeutic. Additionally, there was little to no evidence of side effects associated with the treatment group, aside from mildly altered liver enzyme levels (which could have been an artifact separate from the challenge course) [138].

### 4.3. Post-Exposure Treatments Effective in Small Animal Models – Summarized in Table 4

#### 4.3.1. Mannose-Binding Lectin

Innate immunity is often the first line of defense against invading pathogens. One mechanism by which innate immunity functions relies on the identification of pathogen-associated molecular patterns (PAMPs). PAMPs consist of unique carbohydrate moieties on the external surfaces of foreign microbes, such as hexose and mannose, which are not expressed on the surfaces of the host cells. These PAMPs are then recognized by host proteins such as mannose-binding lectins (MBL), which recognize these high hexose and mannose contents [140]. Filoviruses have large amounts of mannose comprising their glycoproteins and thus are a target of MBL [141]. Upon exposure, host MBL targets filoviruses for compliment-dependent virus neutralization through the lectin pathway of the compliment cascade [142]. When administered in supraphysiological doses before or after lethal challenge with EBOV, recombinant MBL treatment protected 40% of mice [140]. These studies showed that MBL may be a potential post-exposure prophylactic.

**Table 4:** Post-Exposure Treatments Effective in Small Animal Models. Comparison of current treatment candidates at the small animal model level of development, specifically mouse models. Also listed are the afforded levels of protection, the type of drug used to induce immunity and the dosing paradigm used to achieve the listed results.

Drug	Type	Mechanism	Species Tested	Efficacy	Strategy
**Mannose-binding Lectin**	C-type Lectin	Binds to virus and mediates complement-dependent virus neutralization	Mice	40% (EBOV)	350 µg i.p. injection, twice daily for 10 days
**Small-molecule inhibitors**	Compound dependent	Compound dependent	Mice	Compound and dose dependent, ranging from 40%-100% (EBOV and MARV)	Single i.p. injection of 2-5 mg/kg between 1-3 days post-exposure
**Hexamminecobalt (III) Chloride**	Metal ion based drug	Inhibits viral replication	Mice	20% (EBOV)	Daily i.p. injections of 2-8 mg/kg

#### 4.3.2. Small-Molecule Inhibitors

High-throughput screening (HTS) is a significant tool for novel drug discovery. HTS involves screening libraries consisting of thousands to hundreds of thousands of unique molecules against specific targets. Available libraries used in HTS have included natural compounds [143,144], peptides [145], drugs [146], and synthetic compounds [144,147]. Recently, compound FGI-103 was identified during a screen with an EBOV-GFP pseudotyped virus and has shown strong antiviral activity *in vitro *against high doses of EBOV and MARV. FGI-103 was subsequently shown to protect mice against lethal challenges of both EBOV and MARV [148]. Additionally, compound FGI-106 was initially identified in a similar manner and was shown to exhibit strong antiviral activity *in vitro* against EBOV, Rift Valley fever virus (RVFV), all four types of dengue virus (DENV), HCV, and HIV-1. FGI-106 also protected mice against a lethal challenge of EBOV when given post-exposure [149]. Taken together, this suggests that FGI-106 probably acts on a conserved pathway common to these four viruses, and potentially makes for a very intriguing antiviral.

A second screen was done using a collection of 1,990 small molecule compounds obtained from the NCI and the EBOV-GFP pseudotyped virus [150]. As a result, NSC 62914, a reactive oxygen species scavenger, was identified and shown to have high antiviral activity against EBOV, MARV, Lassa virus, RVFV, and VEEV. *In vivo* studies demonstrated that this compound protected mice against a lethal challenge of EBOV and MARV when given either pre- or post-exposure [151].

#### 4.3.3. Hexamminecobalt (III) Chloride

Metal ion-based therapeutics are a new potential class of drugs because they differ from carbon-based compounds due to the charged central ion which determines the molecular geometry of the compound. Through these unique molecular geometries, specific compounds can be isolated that inhibit biological processes, and are unlike traditional carbon-based compounds because of their unique geometry. This difference allows these compounds to form octahedral and square planar molecular geometries. Hexamminecobalt (III) chloride (Cohex) is a complex of a cobalt (III) ion surrounded by six ammonia ligands in a full octahedral coordination. Cohex was initially reported to have antiviral activity against adenovirus and Sindbis virus, and was subsequently thought to have potential broad-spectrum antiviral activities [152]. Cohex was shown to be well-tolerated in mice with no apparent toxicity. Mice were treated with Cohex daily and infected with a lethal dose of EBOV. Cohex-treated mice had a significant increase in mean time-to-death, and the highest concentration treatment group had a 20% survival rate [152]. This suggests that Cohex has the potential to be an effective treatment against EBOV infection. 

### 4.4. Compounds Effective *In Vitro*

#### 4.4.1. Niemann-Pick C1

Niemann-Pick C1 (NPC1), a cholesterol transporter found in endosomes and lysosomes, was recently identified as being required for EBOV replication during a gene trap screen in HAP1 cells using a replication-competent VSV bearing the EBOV glycoprotein (rVSV-GP-EBOV). In these experiments, cells with non-functional NPC1 demonstrated decreased infectivity by rVSV-GP-EBOV; however, expression of a functional NPC1 rescued the normal infectivity of these viruses [20]. NPC1 is known to affect calcium homeostasis as well as endosomal and lysosomal fission and fusion [153,154,155]. It has also been shown to be involved in HIV-1 release [156]. Loss of NPC1 causes a neurological disorder called Niemann–Pick disease, which is characterized by cholesterol accumulation in lysosomes [153]. While heterozygous NPC1 knockout mice (*NPC1*^+/−^) do not show evidence of Niemann-Pick disease, most *NPC1*^+/−^ knockout mice were protected against a lethal challenge of mouse adapted EBOV (80% survival) and MARV (100% survival) [20]. Additionally, small molecules, such as U18666A, have been identified that interfere with NPC1 and cause a cellular phenotype similar to *NPC1* deficiency [157]. As such, U18666A was subsequently shown to block infection of EBOV *in vitro* [21].

#### 4.4.2. HSP-90 Inhibitors

Heat-shock protein 90 (Hsp90) is a molecular chaperone that aids in the folding, trafficking, and proteolytic processing of many proteins [158,159]. As a result of their many functionalities, Hsp90 inhibitors have been designed to combat diseases such as cancer, and there are currently several drugs now in Phase I and II clinical trials [160,161] Additionally, Hsp90 has shown to be important for replication of negative-strand viruses, as well as HCV, HBV, and polio [162,163,164,165]. The effects of several natural and synthetic Hsp90 inhibitors on EBOV replication were tested *in vitro*. Results of this study demonstrated that three Hsp90 inhibitors significantly inhibited the replication of EBOV in Vero cells and primary human monocytes, suggesting their use as a potential therapeutic [158].

#### 4.4.3. Δ-Peptide Immunoadhesins

Ebolaviruses express two secreted glycoproteins, soluble GP (sGP) and small soluble GP (ssGP) [166]. sGP has been associated with stabilization of the endothelial barrier function and reduction of endothelial barrier permeability by opposing the effects of TNF-α. These effects are in direct opposition to the observed roles for GP, which has been associated with endothelial cell destruction [167]. ssGP has yet to have a clear role during infection [166]. Each of the GP forms contains identical N-termini but differ in the structure of their C-termini. During the differentiation process of the C-termini, the homodimer sGP is cleaved by furin to yield the mature sGP and a Δ-peptide which is retained in cells longer as compared to sGP. When these Δ-peptides from EBOV, SUDV, and TAFV were fused with Fc tags and transfected into cells before infection, they were capable of inhibiting both EBOV and MARV infection in a dose dependent fashion [166]. These observations indicated that Δ-peptides might play an important role in filovirus pathogenesis, and could be exploited as a novel anti-filoviral therapeutic [166].

#### 4.4.4. C-Peptides

While many events in filovirus entry remain undiscovered, a fusion mechanism similar to HIV and SARS-CoV is thought to occur such that a conformational rearrangement of glycoproteins on the viral surface results in viral fusion with the cellular membrane. Inhibitors of viral fusion have been developed for HIV-1 and SARS-CoV. These inhibitors prevent the C-terminal heptad repeat in the envelope protein from interacting with the cellular membrane proteins by directly competing and blocking its interaction with the N-terminal heptad repeat, which normally would result in the formation of the six-helix bundle required for membrane fusion. C-peptides, which are inhibitors of viral envelope fusion, have had limited success against filoviruses in the past, most likely due to the suggestive evidence that filovirus fusion occurs far along in the endosomal maturation process [168,169,170,171,172]. However, when these C-peptides were conjugated with an argenine-rich segment of HIV-1’s TAT protein (a protein known for its endosomal localization) the conjugated C-peptide exhibited marked antiviral effects, up to 99% inhibition of EBOV and MARV *in vitro* [172,173,174]. Unfortunately, the concentrations used to generate this inhibition *in vitro* were not possible in the clinical setting, but this report indicates that future C-peptide research may result in filovirus entry prevention for future therapeutics.

#### 4.4.5. Alkylated Porphyrins

A recent report that screened 2,200 molecules demonstrated that chlorophyllide was able to decrease the section of HBV DNA in a HBV antiviral assay. These results were obtained at compound concentrations which exhibited no cytotoxic effects. This molecule is an alkylated porphyrin containing copper and as such this compound is carries a charge at neutral pHs [175]. During these screens, the chlorin e6 compound, a metal-free chlorophyllide-like molecule, was found to be the most potent and was subsequently tested against other viruses, including MARV. During testing, the chlorine e6 compound showed significant antiviral activity *in vitro* against MARV. This compound also inhibited Junin virus, DENV, HCV, and HIV-1 [175].

#### 4.4.6. Benzodiazepine Small Molecule Compounds

Another recent study that examined a library of 52,500 compounds yielded 57 candidates capable of generating ≥90% inhibition of a HIV-1/EBOV-GP pseudotyped virus, while not interfering with the HIV-1/VSV-G control virus. From these candidates, compound 7, a benzodiazepine derivative, showed an ability to inhibit both EBOV and MARV to a similar degree *in vitro *[176]. Results from these experiments suggested that compound 7 acts at an early stage of viral entry, preventing infection by an unknown mechanism [176].

#### 4.4.7. LJ001

LJ001, an aryl methyldiene rhodanine derivative, was identified during a high-throughput screening of inhibitors for Nipah virus entry in the context of a VSV-pseudotyped vector [177]. This compound was subsequently shown to inhibit a variety of enveloped viruses, including MARV, EBOV, Nipah, RVFV, HIV-1, HCV, WNV, *etc*. [177]. However, it did not inhibit nonenveloped viruses such as adenovirus and reovirus. Further testing demonstrated that LJ001 binds to the viral membrane and prevents virus-cell fusion. While initial testing in mice did not show antiviral efficacy, further development of this compound to improve pharmacokinetics and potency is distinctly possible [177].

## 5. Conclusions

Development of medical countermeasures for EBOV and MARV remain a high priority and substantial progress has been made over the past decade. We have moved from the inability to protect from infection in various animal models of disease to a realm of medical countermeasures that protect prophylactically and more recently successful treatments that can be employed following known exposure to the viruses. Initial efforts, focused on preventing the disease with vaccination strategies, ranged from subunit vaccines to VLPs, vectored systems, DNA vaccines, and live-attenuated virus systems that express the EBOV or MARV glycoproteins. To that aim, vaccine efficacy has been achieved by multiple vaccines against parenteral and aerosol routes of exposure. With the success of these new vaccine platforms, the attention of the past 5 years has focused on the ability to treat infected patients. In the animal models, success has been demonstrated with traditional small molecules and antibodies directed against the virus or critical host proteins or pathways associated with pathogenesis. The ability to utilize various RNA silencing technologies has been a focus for therapeutics that could be beneficial for filovirus infection, other infectious diseases and cancer therapy. Despite these successes, there is much work to do to adequately prepare for this infectious threat. The ability to provide a beneficial therapeutic impact at a point when patients experience clinical symptoms and seek relief from caregivers remains a hurdle for the medical countermeasure development. Moreover, the quality of life of patients following infection and treatment may require additional development efforts or the combination of multiple therapeutic approaches. As seen in outbreaks, the clinical sequelae observed in patients that survive infection are severe and life changing. These observations emphasize the need for medical countermeasures that not only provide survival but also decrease morbidity and long-term pathological outcomes following infection. Lastly, the funding resources fortitude and the ability to navigate regulatory pathways will be essential to reaching either emergency use authorization (EUA) status or licensed drug status for therapeutics and vaccines. However, the field remains optimistic that medical countermeasure solutions for human use are possible.
